# The Effects of sodium bicarbonate Ringer’s solution on acute kidney injury and the clinical outcomes after liver transplantation: A randomized controlled trial

**DOI:** 10.3389/fphar.2022.982472

**Published:** 2022-10-06

**Authors:** Hao Wu, Gaige Meng, Chunlong Zuo, Jiang Wang, Shiyun Jin, Lijian Chen, Ye Zhang

**Affiliations:** ^1^ Department of Anesthesiology, the Second Affiliated Hospital of Anhui Medical University, Hefei, China; ^2^ Department of Anesthesiology, the First Affiliated Hospital of Anhui Medical University, Hefei, China

**Keywords:** sodium bicarbonate Ringer’s solution, normal saline, acute kidney injury, clinical outcomes, liver transplantation

## Abstract

**Background:** Acute kidney injury is the most common complication after liver transplantation. Sodium bicarbonate Ringer’s solution is a new type of crystalloid solution that has been recently used in the clinical setting. Whether sodium bicarbonate Ringer’s solution reduces the occurrence of postoperative AKI and improves the clinical outcomes of liver transplantation patients is not clear.

**Objective:** To compare the effects of sodium bicarbonate Ringer’s solution versus normal saline on acute kidney injury and clinical outcomes following classic orthotopic liver transplantation.

**Methods:** Sixty-four participants were randomly assigned to the sodium bicarbonate Ringers (BRS) group or the normal saline (NS) group. The primary outcomes were the incidence and severity of acute kidney injury after liver transplantation. The secondary outcomes included the rate of renal replacement therapy, length of mechanical ventilation, stay in the ICU, stay in the hospital after surgery and 30-day mortality. Other outcomes included the concentration of sodium, chloride, bicarbonate, anion gap, lactate concentration and changes in chloride preoperatively and postoperatively.

**Result:** Sixty-two patients completed the trial and were analyzed, with 31 patients in each group. There was a significantly lower rate of postoperative acute kidney injury in the BRS group (14/31, 45.2%) than in the NS group (24/31, 77.4%), with a relative risk of 0.58 (95% CI, 0.38–0.90; *p* = 0.009). The severity of AKI in the BRS group was lower than that in the NS group (Z = -2.932, *p* = 0.003). There was no significant difference observed in the secondary outcomes. For other outcomes, the concentration of preoperative sodium was lower than postoperative sodium in the NS group (137.2 vs. 140.4, *p* = 0.009). The concentration of preoperative chloride was lower than that of postoperative chloride in the NS group (102.9 vs. 106.2, *p* < 0.001). The change in the concentration of chloride in the BRS group was lower than that in the NS group (1.6 vs. 4.7, *p* = 0.006).

**Conclusion:** Sodium bicarbonate Ringer’s solution reduced the incidence and severity of acute kidney injury after classic orthotopic liver transplantation.

## Introduction

Liver transplantation is the ultimate and effective treatment for end-stage liver disease. More than five thousand liver transplantations are performed in China every year. Acute kidney injury (AKI) is the most common complication after liver transplantation, and the incidence was reported to be 17–95% in different studies (Barri et al., 2009; Saner et al., 2012; Durand et al., 2018). AKI is typically due to a combination of factors, including recipient factors, surgical factors and perioperative factors (de Haan et al., 2017; Durand et al., 2018). Any degree of AKI after liver transplantation is associated with an increased rate of mortality, longer ICU stays, and higher hospital costs (Durand et al., 2018). Therefore, reducing the incidence of AKI in liver transplantation patients is important.

All liver transplantations require intravenous crystalloid solutions for vascular volume repletion and organ perfusion during the surgery. Although sodium lactated Ringer’s solution and normal saline (0.9% sodium chloride) are now the commonly used crystalloid solutions in the clinic, both have limitations. The lactate in sodium lactate Ringer’s solution is mainly metabolized through the liver, which can worsen lactic acidosis and increase the burden of the new liver after transplantation (Murphy et al., 2001; Golse et al., 2019; Takahashi et al., 2019). Previously, normal saline was used in liver transplantation because it did not contain exogenous lactate. However, normal saline has a much higher chloride concentration than human plasma and can cause hyperchloremia in patients during rapid replenishment (Xue et al., 2019). In a retrospective study of liver transplant recipients, the infusion of higher volumes of chloride-liberal fluids and the preoperative status were associated with an increased risk for postoperative AKI (Nadeem et al., 2014). However, in a retrospective study of pediatric liver transplantation patients, it was found that normal saline did not increase the rate of postoperative AKI compared with lactate Ringer’s solution (Dai et al., 2021). Whether normal saline causes AKI after liver transplantation is no longer proven in these retrospective studies, so a prospective randomized controlled trial is needed.

Sodium bicarbonate Ringer’s solution is a new type of crystalloid solution composed of various electrolytes including Na^+^ 130 mmol/L, K^+^ 4.0 mmol/L, Ca^2+^ 1.5 mmol/L, Mg^2+^ 1.0 mmol/L, Cl^−^ 109 mmol/L, HCO_3_^−^ 28 mmol/L and Citrate^3−^ 1.3 mmol/L (Wang et al., 2021). Studies have shown that sodium bicarbonate Ringer’s solution can have a protective effect on renal function and has a promising clinical effect in supplementing the circulating blood volume (Satoh et al., 2005; Ma et al., 2021; Bian et al., 2022; Yu et al., 2022). However, the clinical outcomes of sodium bicarbonate Ringer’s solution in liver transplantation are still unclear because clinical trial data have been rarely reported thus far.

To test the hypothesis that sodium bicarbonate Ringer’s solution reduces postoperative AKI and improves clinical outcomes. We designed a randomized controlled trial to compare the effects of the intraoperative application of sodium bicarbonate Ringer’s solution versus normal saline in patients undergoing classic orthotopic liver transplantation.

## Materials and methods

### Ethical considerations

This study followed the Consolidated Standards of Reporting Trials (CONSORT) reporting guidelines. Ethical approval for this study was provided by the Ethical Committee of First Affiliated Hospital of Anhui Medical University (Ethical Committee No. PJ-2020-01-10). This trial was registered at the Chinese Clinical Trial Registry (ChiCTR1900028158).

### Study design and participants

This study was conducted in the Department of Anesthesiology, the First Affiliated Hospital of Anhui Medical University in Hefei, China. The trial was conducted from January 2020 to March 2022. Sodium bicarbonate Ringer’s solution (H20190021, specification 500 ml; Jiangsu Hengrui Pharmaceutical, China) was used in the sodium bicarbonate Ringer’s (BRS) group. Normal saline (0.9% sodium chloride) (H12020024, specification 500 ml; Guangdong Dazhong Pharmaceutical, China) was used in the normal saline (NS) group. Randomization was performed at a 1:1 ratio using a computer-generated random number table, and then the numbers were concealed in an envelope. Participants were identified from the liver transplantation waiting lists. The inclusion criteria were adults (age >18 years old) and the need for classic orthotopic liver transplantation. The exclusion criteria included preoperative renal replacement therapy, preoperative renal dysfunction (eGFR <60 ml/min/1.73 m^2^), preoperative coma, history of cardiac failure, hypothyroidism or hypermagnesemia.

### Perioperative management

Recipients received standard monitoring, including electrocardiography, heart rate, oxygen saturation, invasive arterial blood pressure, central venous pressure, temperature, urine output, and depth of anesthesia, which were measured by the bispectral index (BIS) or patient state index (PSI). Continuous cardiac index (CI) and stroke volume variation (SVV) were measured by FlowTrac/Vigileo^®^ measurements. Peripheral venous access was established, and tracheal intubation was performed under anesthesia induction with 0.03–0.05 mg/kg midazolam, 0.1–0.3 mg/kg etomidate, 0.5–1 μg/kg sufentanil and 0.2–0.3 mg/kg cisatracurium. Intraoperative anesthesia and muscle relaxation were maintained using propofol, sufentanil, cisatracurium and dexmedetomidine.

From the beginning of anesthetic induction, the participants were treated with 2 ml/kg/h crystalloid solutions. Goal-directed hemodynamic management was used to guide the intravenous crystalloid infusion (Martin et al., 2020; Froghi et al., 2022). When there was no bleeding, 2 ml/kg/h was the background infusion volume. When bleeding occurs during the operation, the intraoperative infusion speed will be accelerated to maintain the circulating volume. When SVV>13% was caused by massive bleeding, 3 ml/kg crystalloid solution was given rapidly within 10 min and repeated until the SVV ≤13%. At the same time, blood transfusion was conducted according to the transfusion guidelines. If the mean arterial blood pressure was ≤65 mmHg or 20% lower than the baseline value, norepinephrine was infused. If the arterial blood pressure was 20% higher than the baseline value, sufentanil 0.1–0.2 μg/kg was injected. If it was ineffective, nicardipine 0.3 mg was injected.

The perioperative patient blood management followed the guidelines of the China, transfusion of whole blood and blood components WS/T 623-2018. When hemoglobin was lower than 70 g/L, a red blood cell suspension was infused to maintain hemoglobin ≥70 g/L. When the prothrombin time (PT) or activated partial thromboplastin time (APTT) was greater than 1.5 times the normal value, fresh frozen plasma was infused. If the platelet count was <50 × 10^9^/L, the patient was given a platelet transfusion, and if the plasma fibrinogen was <1 g/dl, the patient received an infusion of cold precipitation. Albumin was infused when albumin was lower than 30 g/L. Autologous blood reinfusion technology was used when necessary.

After the completion of surgery, all patients were transferred to the ICU for on-going monitoring and postoperative care and were extubated according to standardized ICU protocols. When it was necessary to infuse crystalloid solution in the ICU, all patients were given normal saline. Norepinephrine and terlipressin were used to maintain arterial pressure.

### Study outcomes

The primary outcomes were the incidence and severity of AKI after surgery. AKI was defined according to the “Kidney disease: Improving Global Outcomes” (KDIGO) system. The severity of AKI was staged according to the KDIGO system within 2 days after surgery.

The secondary outcomes included the rate of renal replacement therapy, length of mechanical ventilation, length of stay in the ICU, length of stay in the hospital after surgery, and 30-day mortality.

Other outcomes included the concentration of sodium, chloride, bicarbonate, anion gap, lactate concentration and changes in chloride preoperatively and postoperatively. Preoperative refers to the results measured within 24 h before the operation. Postoperative refers to the results measured at the end of surgery or within 1 h after the patients were sent to the ICU. The anion gap was calculated as (Na^+^) − (Cl^−^+ HCO_3_^−^). The change in the concentration of chloride was calculated as the postoperative chloride minus the preoperative chloride.

### Statistical analysis

The incidence of AKI was used for the sample size calculation. To achieve significance, we calculated that a sample of 64 patients would be required for a reduction in the incidence of AKI from 83% in the NS group to 50% in the BRS group according to our pre-experiment (accounting for a two-sided α-level of 0.05, a statistical power of 80%, and allowing for a loss to follow-up of 10%).

The data were analyzed using IBM SPSS Statistics for Windows version 20.0. Continuous variables are presented as the means and standard deviations (SDs) or medians and interquartile ranges (IQRs: 25–75%) and were compared with independent t tests (parametric) or Mann–Whitney *U* tests (nonparametric). Categorical variables were reported as frequencies and proportions and analyzed via the Pearson χ^2^ test or Fisher’s exact test, as appropriate. *p* < 0.05 was considered statistically significant.

## Results

### Baseline characteristics

**FIGURE 1:**
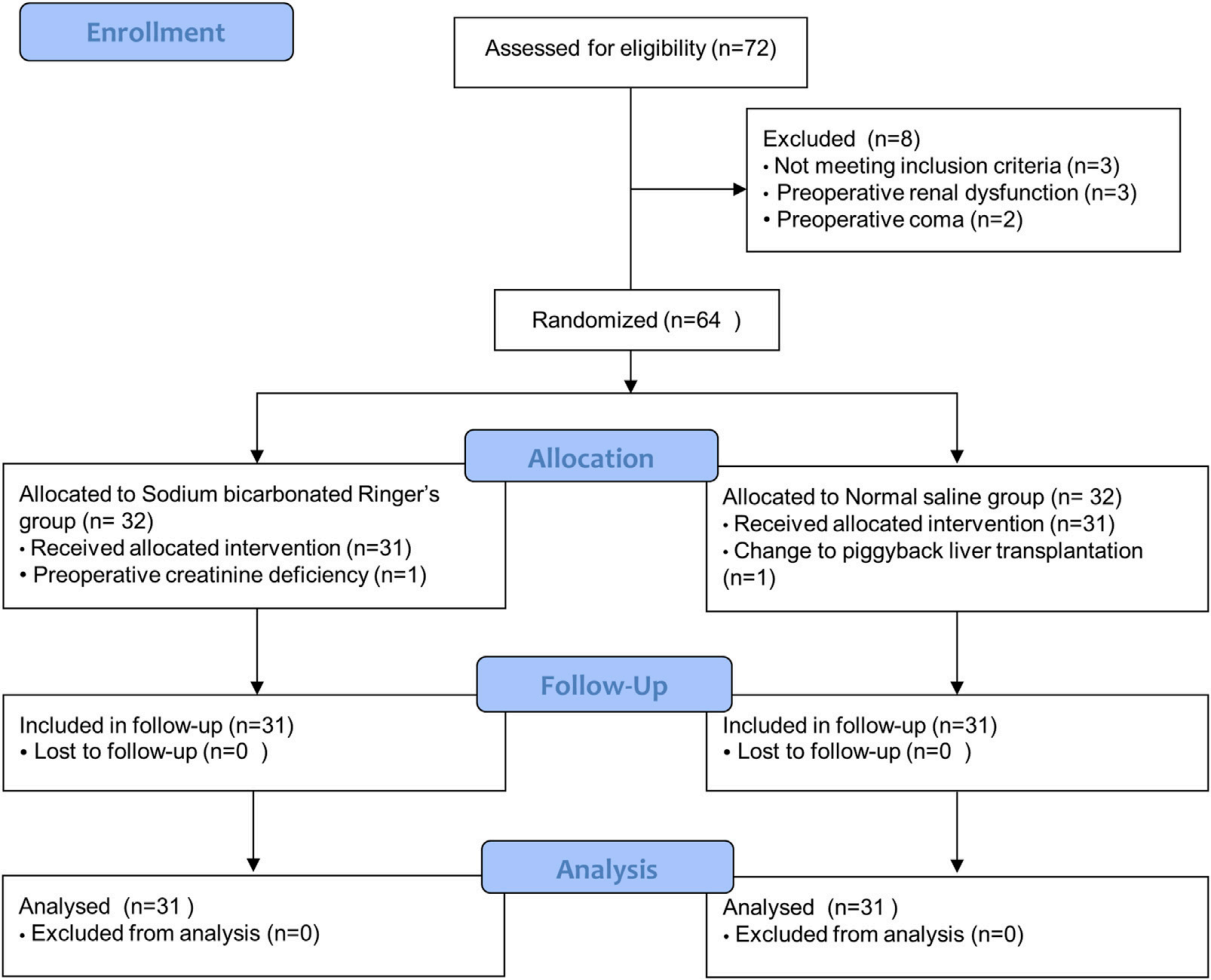
Flow diagram.

A total of 72 patients were screened for eligibility, and 64 patients were ultimately enrolled and randomized. One patient in the BRS group was found to lack preoperative creatinine after randomization. One patient in the NS group was changed from classical orthotopic liver transplantation to piggyback liver transplantation. The study finally included 62 patients. There were 31 patients in the bicarbonate Ringers group and 31 patients in the saline group (Figure 1). The baseline characteristics were similar between the two groups (Table 1).

**TABLE 1:** Baseline characteristics of the patients.

Variable	Normal Values	BRS (n = 31)	NS (*n* = 31)	*p*-value
Age, years		47.4 ± 9.8	47.6 ± 10.8	0.922ϕ
Sex; male, n (%)		25 (80.6)	24 (77.4)	0.755ϕ
Weight, kg		64.0 ± 11.3	65.3 ± 10.3	0.641ϕ
Body mass index, kg/m^2^	18.5–23.9	22.7 ± 3.3	23.0 ± 3.2	0.787ϕ
MELD score	6-40	16 (13-19)	17 (13-25)	0.554¥
Creatinine, μmol/L	58.0–111.0	63.1 ± 18.0	61.4 ± 14.1	0.675ϕ
eGFR, mL/(min·1.73m^2^)	>90	115.9 ± 20.3	116.9 ± 15.7	0.824ϕ
Hemoglobin, g/L	120–160 male	101.0 ± 31.3	101.0 ± 21.7	0.996ϕ
	110-150 female			
Albumin, g/L	35-50	36.3 ± 7.3	34.3 ± 6.8	0.268ϕ
Glucose, mmol/L	3.9–6.1	6.2 ± 2.1	5.4 ± 1.6	0.090ϕ
MAP, mmHg	70–105	89.4 ± 13.7	87.5 ± 14.5	0.584ϕ

Data are the mean ± SD, or median (interquartile range: 25–75%). ϕ Independent *t* test was used. ¥ Mann–Whitney *U* test was used. MELD, model for end-stage liver disease; eGFR, estimated glomerular filtration rate; MAP, mean arterial pressure. n, number of patients.

### Intraoperative and ICU factors

The intraoperative factors, including the duration of surgery, duration of anhepatic phase, crystalloid infusion volume, blood product requirements, urine output, hemodynamics and requirements for norepinephrine, were similar for the two groups. However, the infusion volume of 5% sodium bicarbonate in the BRS group was lower than that in the NS group (100 vs. 150, *p* < 0.05) (Table 2).

**TABLE 2:** Intraoperative and ICU factors in the two groups.

Variable	BRS (*n* = 31)	NS (*n* = 31)	*p*-value
Intraoperative			
Duration of surgery, minutes	393.5 ± 57.4	410.0 ± 73.7	0.330ϕ
Duration of anhepatic phase, minutes	53.7 ± 12.0	50.3 ± 8.3	0.198ϕ
Crystalloid infusion volume, mL	2483.9 ± 701.0	2516.1 ± 779.8	0.865ϕ
5% sodium bicarbonate, mL	100 (50-150)	150 (100-200)	0.041¥
Intraoperative cell salvage, mL	0(0-0)	0(0-361)	0.687¥
Red blood cells, units	4(0–9.5)	5(4-8)	0.437¥
Fresh frozen plasma, mL	1000(800-1800)	1000(950-1250)	0.798¥
20% Human albumin, g	100.0 ± 47.1	90.8 ± 33.2	0.378ϕ
Urine output, mL	1509.7 ± 740.5	1633.9 ± 630.8	0.480ϕ
Dosage of norepinephrine, mg	2(1-4)	2.5(1.4–4.2)	0.698¥
Hemodynamics			
MAP at skin incision, mmHg	74.5 ± 10.1	72.3 ± 10.1	0.403ϕ
MAP at the beginning of anhepatic phase, mmHg	79.0 ± 13.4	77.3 ± 13.8	0.623ϕ
MAP at the end of anhepatic phase, mmHg	82.7 ± 13.2	81.4 ± 14.9	0.727ϕ
MAP at the end of surgery, mmHg	74.5.4 ± 11.0	72.4 ± 11.1	0.458ϕ
Duration of MAP<65 mmHg, min	20(5-65)	35(15-60)	0.498¥
Duration of MAP<60 mmHg, min	5(0-15)	5(0-15)	0.895¥
Duration of MAP<50 mmHg, min	0(0-0)	0(0-0)	0.701¥
ICU			
Red blood cells, units	3(0-5)	2(0-4)	0.676¥
Fresh frozen plasma, mL	0(0-400)	0(0-0)	0.425¥
Infusion volume, mL/h	170.7 ± 28.7	167.5 ± 34.8	0.692ϕ
Urine output, mL/h	151.9 ± 47.7	150.9 ± 46.1	0.929ϕ
Dosage of norepinephrine, mg	0(0-6)	0(0-12)	0.646¥
Dosage of terlipressin, mg	2(1-2)	2(1-2)	0.891¥

Data are the mean ± SD, or median (interquartile range: 25–75%). ϕ Independent *t* test was used. ¥ Mann–Whitney *U* test was used. MAP, mean arterial pressure.

The ICU factors, including blood product requirements, infusion volume, urine output, requirements for norepinephrine and terlipressin, were similar for the two groups (Table 2).

### Primary outcomes

The incidence of AKI occurred in 45.2% (14/31) of the patients in the BRS group versus 77.4% (24/31) of the normal saline patients, with a relative risk of 0.58 (95% CI, 0.38–0.90; *p* = 0.009). In the BRS group, AKI stage 1 occurred in eight patients (25.8%), stage 2 occurred in five patients (16.1%), stage 3 occurred in one patient (3.2%), and 17 patients (54.8%) did not have AKI. In the NS group, AKI stage 1 occurred in nine patients (29.0%), stage 2 occurred in nine patients (29.0%), stage 3 occurred in six patients (19.4%), and seven patients (22.6%) did not have AKI. The severity of AKI in the BRS group was lower than that in the NS group (Z = -2.932, *p* = 0.003) (Table 3).

**TABLE 3:** Primary and secondary outcomes.

Variable	BRS (n = 31)	NS (*n* = 31)	*p*-value
Primary outcomes			
Incidence of AKI, n (%)	14 (45.2)	24 (77.4)	0.009 $
Severity of AKI			
No AKI, n (%)	17 (54.8)	7 (22.6)	0.003¥
Stage 1, n (%)	8 (25.8)	9 (29.0)	
Stage 2, n (%)	5 (16.1)	9 (29.0)	
Stage 3, n (%)	1 (3.2)	6 (19.4)	
Secondary outcomes			
Renal replacement therapy, n (%)	0 (0)	2 (6.5)	0.492#
Length of mechanical ventilation, hours	14(9-16)	16(11-26)	0.089¥
Length of stay in the ICU, hours	35(28-43)	36(29-50)	0.525¥
Length of stay in the hospital after surgery, days	23.1 ± 9.4	27.2 ± 11.6	0.142ϕ
30-day mortality, n (%)	1(3.2)	3(9.7)	0.612#

Data are the number of patients (percentages), median (interquartile range: 25–75%) or mean ± SD. $ Pearson χ ^2^ test was used. ¥ Mann–Whitney *U* test was used. ϕ Independent *t* test was used. and # Fisher’s exact test was used. AKI, acute kidney injury. n, number of patients.

### Secondary and other outcomes

There was no significant difference between the groups in the rate of renal replacement therapy, the length of mechanical ventilation, the length of stay in the ICU, the length of stay in the hospital after surgery or 30-day mortality (Table 3).

**FIGURE 2:**
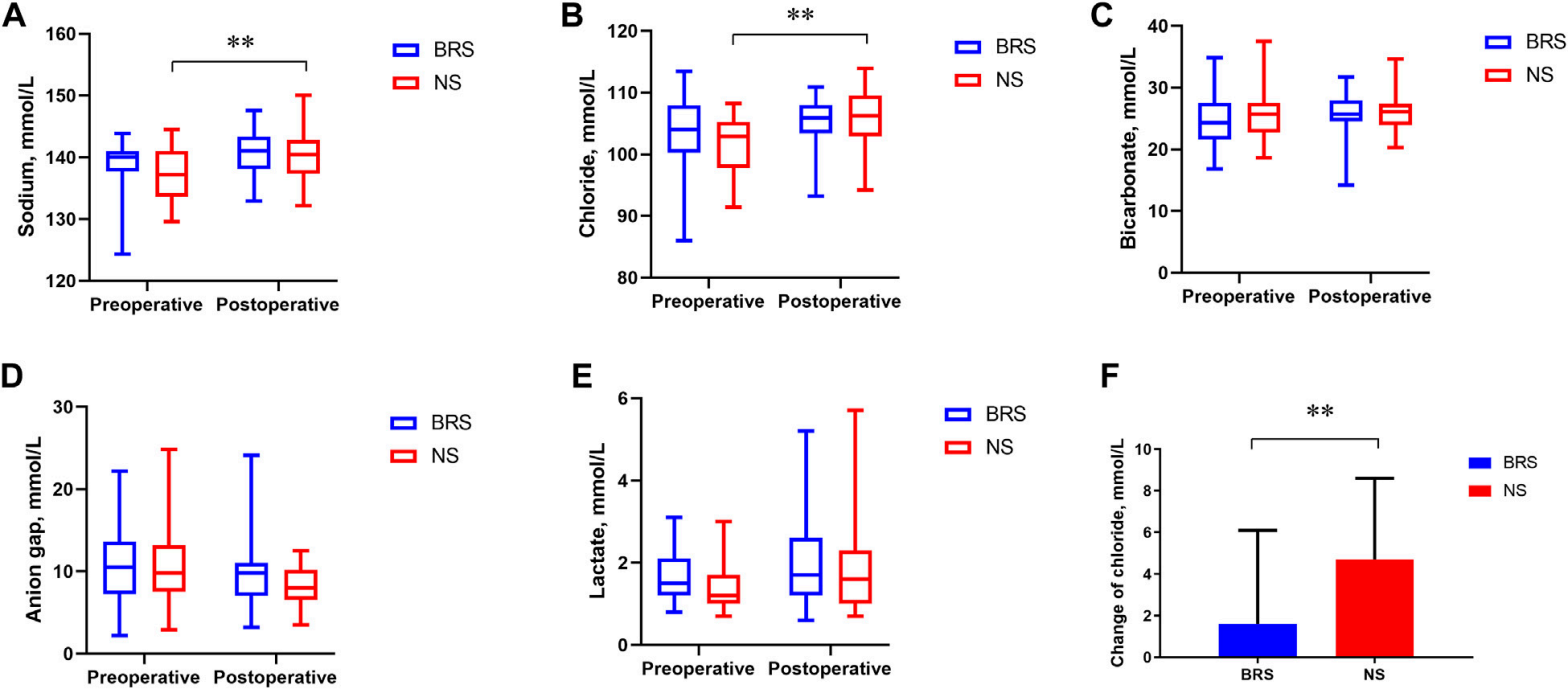
The concentrations of sodium, chloride, bicarbonate, anion gap, lactate and the change in the concentration of chloride preoperatively and postoperatively. The median and interquartile range of sodium **(A)**, chloride **(B)**, bicarbonate **(C)**, anion gap **(D)**, lactate **(E)** and the change in chloride **(F)** preoperatively and postoperatively are shown for the patients in each group. The concentration of preoperative sodium was lower than that of postoperative sodium in the NS group (137.2 vs. 140.4, *p* = 0.009). The concentration of preoperative chloride was lower than that of postoperative chloride in the NS group (102.9 vs. 106.2, *p* < 0.001). The Mann–Whitney *U* test was used in **(A,B)**.The change in the concentration of chloride in the BRS group was lower than that in the NS group (1.6 vs. 4.7, *p* = 0.006). An independent *t* test was used in **(F)**.

The concentration of preoperative sodium was lower than that of postoperative sodium in the NS group (137.2 vs. 140.4, *p* = 0.009). The concentration of preoperative chloride was lower than that of postoperative chloride in the NS group (102.9 vs. 106.2, *p* < 0.001) (Figures 2A,B). The change in the concentration of chloride in the BRS group was lower than that in the NS group (1.6 vs. 4.7, *p* = 0.006) (Figure 2F). There was no significant difference between the two groups in the concentrations of sodium, chloride, bicarbonate, anion gap, and lactate preoperatively and postoperatively. There was no significant difference between the preoperative and postoperative concentrations of sodium, chloride, bicarbonate, anion gap and lactate in the BRS group.

## Discussion

In our study, sodium bicarbonate Ringer’s solution reduced the incidence and severity of AKI after classic orthotopic liver transplantation. There was a significantly lower rate of AKI in the BRS group (45.2%) than in the NS group (77.4%). The severity of AKI in the BRS group was also lower than that in the NS group. Four patients died within 30 days after the operation. One patient in the BRS group developed intracerebral hemorrhage 3 days after the operation. One patient in the NS group had a massive hemorrhage 7 days after the operation. Two patients in the NS group had multiple organ dysfunction syndrome 4 and 15 days after the operation. There was no difference in 30-day mortality between the two groups. Two patients needed renal replacement therapy, both of whom were in the NS group. The length of mechanical ventilation, stay in the ICU, and stay in the hospital after surgery were shorter in the BRS group than in the NS group, but there was no significant difference. It may be that the sample size was calculated with AKI as the primary outcome. Whether there are differences in the secondary outcomes between the two groups needs to be confirmed by a large sample study.

At present, crystalloid infusion during liver transplantation mainly includes sodium lactate Ringer’s solution, sodium acetate Ringer’s solution, sodium bicarbonate Ringer’s solution and normal saline. However, the lactate in lactate Ringer’s solution and acetate in acetate Ringer’s solution are mainly metabolized through the liver and kidney. The metabolism of liver transplantation patients is slow due to liver dysfunction, which can aggravate the inherent lactic acidosis and increase the burden of the new liver after transplantation (Murphy et al., 2001; Golse et al., 2019; Takahashi et al., 2019). In addition, the blocking of the inferior vena cava in the anhepatic phase and the application of norepinephrine produce lactate. Extensive use of lactate Ringer’s solution and acetate Ringer’s solution may interfere with the diagnosis and judgment of the disease during surgery (Hadimioglu et al., 2008). Therefore, neither of them is ideal crystalloids for liver transplantation patients. Sodium bicarbonate Ringer’s solution, as a relatively newly emerging balanced solution, has been widely used in clinical practice in recent years. Its remarkable advantage is that bicarbonate is completely independent of liver metabolism, and only 10% is excreted through the kidney, which has little impact on renal function. Moreover, compared with other crystal solutions, sodium bicarbonate Ringer’s solution is closer to the components of plasma, which can not only effectively supplement the circulating blood volume but also correct metabolic acidosis. As bicarbonate can directly dissociate from sodium bicarbonate without metabolic processes, it is considered that sodium bicarbonate Ringer's solution is an ideal crystalline solution for patients with liver and kidney dysfunction in the clinic.

Several prospective randomized controlled studies have shown that the application of normal saline is related to acute kidney injury (Self et al., 2018; Semler et al., 2018). However, recent studies found that normal saline did not increase the rate of acute kidney injury (Maheshwari et al., 2020; Finfer et al., 2022). The different results may be due to the different types of patients and surgeries. Patients with normal renal function have good renal function reserve. However, patients with liver transplantation have reduced renal function reserve due to disease, while creatinine increases significantly only when the renal function reserve is exhausted. AKI after liver transplantation is common, with an incidence ranging from 17 to 95% (Barri et al., 2009; Durand et al., 2018). AKI is typically due to a combination of factors, including the recipient, the donor and surgical events (Durand et al., 2018). In particular, normal saline results in reductions in renal blood flow velocity and renal cortical tissue perfusion (Chowdhury et al., 2012). The inferior vena cava should be blocked during the operation, which will affect the blood reflux of the renal vein. Therefore, we should pay more attention to the protection of renal function during classic orthotopic liver transplantation.

In our study, the higher incidence of AKI in the NS group may be related to the higher chloride in normal saline. The concentration of chlorine in normal saline is 154 mmol/L, while the concentration of chlorine in sodium bicarbonate Ringer’s solution is 109 mmol/L, which is closer to the plasma concentration of chlorine. The concentration of preoperative chloride was lower than that of postoperative chloride in the NS group (Figure 2B). The change in the concentration of chloride in the NS group was 4.7 mmol/L, which was higher than that in the BRS group (Figure 2F). The intraoperative infusion of 5% sodium bicarbonate in the BRS group was less than that in the NS group. However, there was no difference in the preoperative and postoperative bicarbonate between the two groups. This may be related to the HCO3^-^ buffer system in the sodium bicarbonate Ringer’s solution (Wang et al., 2021), bicarbonate can be metabolized to carbon dioxide and excreted through the breath. Infusing 8.4% sodium bicarbonate during liver transplantation did not decrease the incidence of AKI (Weinberg et al., 2016). The reason for the lower AKI in the BRS group was that sodium bicarbonate Ringer’s solution is closer to the plasma composition, not sodium bicarbonate in sodium bicarbonate Ringer’s solution.

There are some limitations in this study. As the product packaging of the two crystalloid solutions was quite different, this study was a single-blind randomized study, but the primary outcome was an objective variable not amenable to manipulation. The anesthetics, antibiotics, hormones and other drugs need to be dissolved and diluted in the crystalline solution. However, the compatibility and stability of these drugs in sodium bicarbonate Ringer’s solution are not clear. To ensure safety, the drugs were dissolved and diluted in normal saline. Therefore, a small amount of normal saline was injected into the patients in the sodium bicarbonate Ringer’s solution group during the surgery.

In conclusion, compared to normal saline, sodium bicarbonate Ringer’s solution reduced the incidence and severity of AKI after classic orthotopic liver transplantation.

## Data Availability

The original contributions presented in the study are included in the article/supplementary material further inquiries can be directed to the corresponding author.
